# Electrically Tunable Lens (ETL)-Based Variable Focus Imaging System for Parametric Surface Texture Analysis of Materials

**DOI:** 10.3390/mi13010017

**Published:** 2021-12-23

**Authors:** Jorabar Singh Nirwan, Shan Lou, Saqib Hussain, Muhammad Nauman, Tariq Hussain, Barbara R. Conway, Muhammad Usman Ghori

**Affiliations:** 1 Department of Pharmacy, School of Applied Sciences, University of Huddersfield, Huddersfield HD1 3DH, UK; jorabar.nirwan@hud.ac.uk (J.S.N.); b.r.conway@hud.ac.uk (B.R.C.); 2 EPSRC Future Metrology Hub, University of Huddersfield, Queensgate, Huddersfield HD1 3DH, UK; s.lou@hud.ac.uk; 3 Department of Mathematics and Physics, Texas A&M International University, Laredo, TX 78041, USA; saqib.hussain@tamiu.edu; 4 Division of Mathematical and Physical Sciences, Institute of Science and Technology, 3400 Klosterneuburg, Austria; nouman.kakakhail@gmail.com; 5 System Engineering Department, Military Technological College, Muscat 111, Oman; tariq.hussain@mtc.edu.om; 6 The Wolfson Centre for Bulk Solid Handling Technology, University of Greenwich, London SE10 9LS, UK

**Keywords:** surface texture, electrically tunable lens, materials, hypromellose, surface topography, surface roughness, pharmaceutical tablet, variable focus imaging

## Abstract

Electrically tunable lenses (ETLs) are those with the ability to alter their optical power in response to an electric signal. This feature allows such systems to not only image the areas of interest but also obtain spatial depth perception (depth of field, DOF). The aim of the present study was to develop an ETL-based imaging system for quantitative surface analysis. Firstly, the system was calibrated to achieve high depth resolution, warranting the accurate measurement of the depth and to account for and correct any influences from external factors on the ETL. This was completed using the Tenengrad operator which effectively identified the plane of best focus as demonstrated by the linear relationship between the control current applied to the ETL and the height at which the optical system focuses. The system was then employed to measure amplitude, spatial, hybrid, and volume surface texture parameters of a model material (pharmaceutical dosage form) which were validated against the parameters obtained using a previously validated surface texture analysis technique, optical profilometry. There were no statistically significant differences between the surface texture parameters measured by the techniques, highlighting the potential application of ETL-based imaging systems as an easily adaptable and low-cost alternative surface texture analysis technique to conventional microscopy techniques.

## 1. Introduction

Surface texture may be defined as the repetitive or random deviation from the nominal surface that forms the three-dimensional topography of the surface and includes roughness, waviness, lay, and flaws [1]. In general, roughness is caused by fluctuations in the surface of short wavelengths and is characterised by peaks and valleys of varying amplitudes and spacings. Waviness (macroroughness) is the surface irregularity of longer wavelengths and includes all irregularities whose spacing is greater than the roughness wavelengths. The principal direction of the surface pattern is referred to as lay and is predominately determined by the fabrication method, while flaws are unexpected, unwanted, and unintentional interruptions in the texture [1,2]. These surface components play a vital role in determining the functionality and behaviour of materials used in multiple applications including mechanical components, construction material, surgical implants, and solid pharmaceutical formulations; hence, the measurement and control of these properties is necessary for safe and effective functioning [3,4].

Current surface texture analysis techniques can be divided into two broad categories: a contact type in which during measurement a component of the measurement instrument contacts the surface to be measured and includes contact profilometers and atomic force microscopy (AFM); and a non-contact type which includes coherence scanning interferometry, confocal microscopy, and scanning laser microscope [2]. These techniques have been employed extensively in industry and academia with AFM currently considered the most powerful and versatile technique for nanoscale imaging and multiple studies have successfully employed this technique, either alone or in combination with another imaging or analytical technique, to quantitatively assess the surface attributes [3,4,5,6,7]. However, along with other contact-type techniques, this may cause damage to the sample surfaces and precise attention must be given for the normal loads to be low enough so that the contact stresses do not exceed the hardness of the surface to be measured [2]. Furthermore, measurement of samples can be time consuming, and although this may be improved with the use of non-contact techniques, these systems are generally large, expensive, and require high power consumption. Another drawback of currently employed surface texture analysis techniques is their inability to capture the full surface of a sample; thus, leaving users restricted to being able to analyse only a limited area. Though, to an extent digital holographic microscopy has mended this gap [8,9,10,11,12]; however, this imaging technique is expert and resource intensive with heavy computational load, which necessitates development of an accurate and sensitive numerical reconstruction algorithm for meaningful data processing and results [13]. Moreover, the coexistence of twin images is a continuous challenge with these imaging systems [14,15,16].

A unique solution to the aforementioned drawbacks of conventional surface texture analysis techniques may be found in electrically tunable lenses (ETL), which possess the ability to alter their optical power in response to an electric signal and have an imaging system integrated with an advanced image stitching algorithm [3,17,18]. The mechanism of ETL is achieved via two main approaches: local variations in refractive index which include liquid crystals, and controlling the shape of the lens with the two main techniques including electrowetting and shape-changing polymers [19]. In general, liquid crystal lenses display larger focal length changes with lower applied voltage, although they suffer from chromatic aberration. Electrowetting lenses have the characteristics of large power range and fast response speed, while requiring a high driving voltage and possessing an aperture of limited size [18,19]. Meanwhile, polymer-based liquid lenses allow the flexible selection of aperture size and are composed of an optical fluid and an elastomeric polymer membrane, usually polydimethylsiloxane (PDMS) [18,19,20]. These properties of ETL provide them with multiple unique advantages over other systems including greater compactness due to the use of less optomechanical components, a fast frequency response, less mechanic motion, and lower power consumption [17,18,21,22].

Furthermore, ETL are not only able to image the areas of interest but are also able to obtain different spatial depth perception (depth of field, DOF) [19,23,24,25,26]. Hu et al. [19] tackled the issue of shallow working volume caused by the limited DOF of conventional lenses by implementing an ETL to enlarge the 3D shape measurement range. With this approach, the authors were able to capture always in-focus phase-shifted fringe patterns by precisely synchronising the tunable lens attached to a camera with the image acquisition and the pattern projection. A prototype system was developed which was able to perform high-quality measurement for a depth range of approximately 1000 mm (400–1400 mm) and a measurement error of 0.05%. The authors concluded that such a system is suitable to be used for real-time 3D shape measurements by experimentally measuring moving objects.

A practical application of ETL was explored by Fahrbach et al. [23] who combined a light-sheet microscope with an ETL for the in vivo investigation of highly dynamic biological samples, for example, a beating zebrafish heart. The authors state that in order to obtain images of different planes using a conventional light-sheet microscope, it is necessary to move the sample relative to the fixed focal plane of the detection objective lens which limits speed, precision, and may be harmful to the sample. However, with the use of ETL, the authors were able to achieve flexible volume imaging at much higher speeds than previously reported and the DOF allowed them to image 17 planes within a beating zebrafish heart [19]. Similarly, a study conducted by Chen and Lin [26] demonstrated an electrically tunable endoscopic system by adopting a LC lens with a large spatial depth perception. As conventional endoscopic systems consist of several solid lenses and suffer from a fixed and limited DOF, the authors were able to image the object at different objective distances using the new system and the corresponding DOF helped in enlarging the total spatial depth perception in the scene [27].

However, although the usefulness of DOF and controlling the optical power in response to the electrical signal has been reported, we hypothesised these features can be exploited to investigate the parametric surface texture analysis of the materials. It is anticipated that the DOF in ETL will be able to provide accurate measurement of peaks and valleys present on the surface of materials thus allowing quantification of surface texture parameters. Hence, the aim of the present study was to develop and utilise a low-cost non-destructive ETL-based imaging system for 3D surface characterisation of materials. To ensure efficient and accurate analysis of the material surface, the system will firstly be calibrated using a widely adopted focus measure. The performance of the newly developed system will also be compared and comprehensively validated against a previously validated surface texture analysis technique—optical profilometry. Moreover, an image stitching feature [3] will also be integrated to demonstrate its ability to analyse the complete surface of the model material sample, a pharmaceutical tablet formulation. The surface properties of solid-state pharmaceutical formulations are of critical importance in determining the performance of the dosage form. The surface of dosage forms such as tablets, can be modified, either intentionally, for example by coating, or unintentionally through processing. A matrix tablet in its simplest form is a blend of a drug and a polymer that controls the rate of drug release and in current study this was the pharmaceutical system used as a model material. 

## 2. Materials and Methods

Hydroxypropyl methylcellulose, HPMC, (K4M) and propranolol HCl were acquired from Colorcon, Dartford, U.K. and Sigma-Aldrich Chemical Co., St. Louis, MI, USA, respectively.

### 2.1. Electrically Tunable Lens (ETL)-Based Imaging Setup

**Figure 1:**
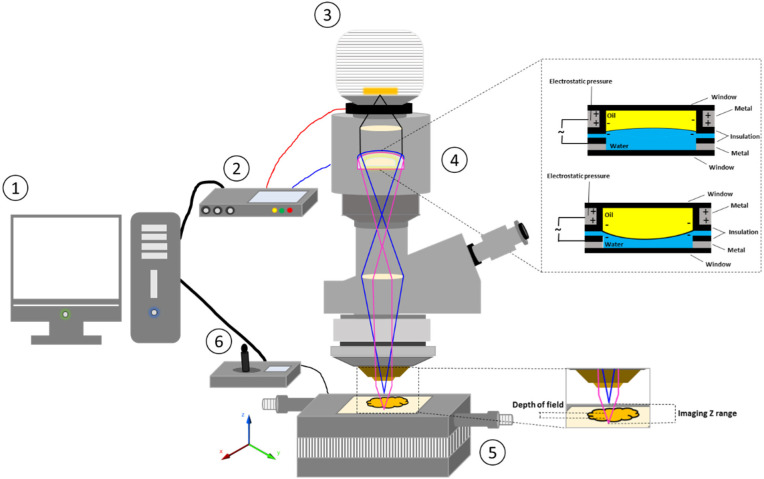
Schematic illustration of ETL-based imaging system. (1) computer; (2) ETL driver; (3) camera; (4) ETL; (5) automatic motorized vertical translation stage; and (6) stage controlling system.

The multiple components of the ETL-based imaging system are displayed in Figure 1. The system comprised a polymer-based liquid ETL (Phaseview, Verrières-le-Buisson, France) attached to a driver (Phaseview, Verrières-le-Buisson, France) which provided a voltage to the ETL and allowed tuning of this voltage (voltage range) enabling adjustment of the ETL focal power (focal power range). The ETL driver was connected to a computer used to control the voltage. Images were captured via a camera attached to the ETL system which relayed the live images to the computer for image processing to obtain 3D surface texture parameters. In the current imaging system, a charge-coupled device (CCD) camera with a light-emitting diode (light source) was employed. Furthermore, four objective lenses were available to be used with the system possessing magnification powers of 5×, 10×, 20×, and 50×. The specifications of the objective lenses are displayed in Table 1. As the focus of the present research was DOF which decreases, along with z-resolution, as numerical aperture increases, the objective lens utilised for the analyses in this study was 5× magnification. Moreover, to secure the sample under analysis, a high precision 0.25 µm automatic motorised, computer controlled, vertical translation stage was used.

### 2.2. System Calibration

Calibration of the ETL imaging system was conducted using a high precision 0.25 µm automatic motorised vertical translation stage. Briefly, this stage was set to different focus heights separated by regular intervals of 0.25 µm. For each focus height (µm), a series of images was captured while modifying the focal length setting by adjustment of the control current (mA) applied to the ETL and a focus measure was computed for each image using the Tenengrad operator (Equation (1)) [28,29]. Although multiple focus measure techniques are available, an ideal focus measure function produces its maximum value at the plane of best focus and values decrease in both directions as the defocus measure increases. This function produces a bell-shaped curve when focus measure values are plotted against image sequences with different focus distances. In this regard, the Tenengrad operator has been proven to be more accurate, robust, and less sensitive to noise than other focus measure functions [17,18,19].

(1) $$\phi TEN\left(I\left(x,y\right)\right)=\underset{M}{\sum }\underset{N}{\sum }{\left[S\left(x,y\right)\right]}^{2}\;$$

where,

(2) $$S\left(x,y\right)=\sqrt{|G_{x}\left(x,y\right){|}^{2}+|G_{y}\left(x,y\right){|}^{2}}$$

where *G_x_* (*x*, *y*) and *G_y_* (*x*, *y*) are the results of the Sobel operator which is used as an edge detection method, applied to the area of interest, *I*, along the *x* and *y* direction, respectively. The focus measure results were plotted against the corresponding control current (mA) applied which resulted in Gaussian-like curves with the peak representing the plane of best focus. To ensure the focus measure accurately detects the plane of best focus, the control current (mA) required to reach the greatest focus measure (a.u.) for each focus height (µm) (peak of each curve) was plotted against its corresponding focus height to establish a linear relationship.

### 2.3. Preparation of Pharmaceutical Tablet Formulation

A pharmaceutical tablet formulation (model sample material) was prepared as described by Nirwan et al., 2019 [3]. Briefly, a powder mixture of HPMC (30% *w/w*) and propranolol (70% *w/w*) was prepared using a tubular mixer (Artisan Technology Group^®^, Kansas City, MO, USA) at 50 rpm for 15 min, followed by content uniformity testing of the mixture using ultraviolet spectroscopy which confirmed uniform mixing. Tablets of 300 mg of the powder mixture were prepared using a Testometric™ M500–50 CT (Testometric™ Company Ltd., Rochdale, U.K.) apparatus directly connected to a computer with a 50 kN load cell equipped with a 13.00 mm Atlas Evacuable Pellet Die (Specac^®^ Limited, Orpington, U.K.). Polished flat-faced punches were used, where the lower punch was held stationary while the upper punch moved at a speed of 3 mm min^−1^ during loading and 1 mm min^−1^ on unloading and an applied pressure of 150 MPa was used. Following ejection of the tablets, they were stored in an air-tight container over silica gel for 24 h to allow for elastic recovery before further investigation. All compaction work was conducted at a relative humidity and temperature in the range of 20% to 35% RH and 25 °C to 27 °C, respectively.

### 2.4. Imaging and Parametric Surface Texture Analysis of Matrix Tablet

**Figure 2:**
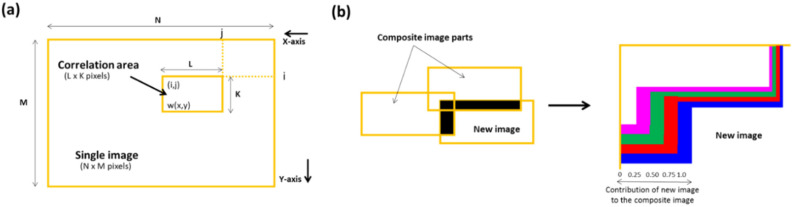
Schematic illustrations showing (**a**) image stitching and (**b**) blending of overlapping image intensities. This image is adopted from [3] with permission from the publisher, Royal Chemical Society.

Each tablet was fixed on an automatic motorised vertical translation stage using a carbon sticky dot. Following this, 35 nanoscale images were captured to allow analysis of the whole tablet surface using the ETL system in a controlled environment (temperature 18–25 °C and RH 25–33%) with an acquisition time of <1 min. The 35 individual images were stitched together to produce a single image revealing the overall tablet surface by implementing an image stitching algorithm using MATLAB^®^ 2017 software (The MathWorks, Inc., Natick, MA, USA). The overall process consisted of two stages: (a) image stitching and (b) image blending. In the first stage, a relative position of the collected images was generated followed by the identification of the correlation point which was performed by sliding the adjacent edges of the image in both directions until a best match of edge features was achieved. This method involves the choice of an optimal search space as illustrated in Figure 2a, in which a detailed assessment was performed to identify the best correlation (which was a 20% overlap in the present study). The normalised cross-correlation can be expressed by the following equation, Equation (3):

(3) $$\frac{\sum_{x=0}^{L-1}\sum_{y=0}^{K-1}\left(w\left(x,y\right)-w\right)\left(f\left(x+i,y+j\right)-f\left(i,j\right)\right)}{\sqrt{\sum_{x=0}^{L-1}\sum_{y=0}^{K-1}{\left(w\left(x,y\right)-w\right)}^{2}}\;\sqrt{\sum_{x=0}^{L-1}\sum_{y=0}^{K-1}{\left(f\left(x+i,y+j\right)-f\left(i,j\right)\right)}^{2}}}$$

where *w*(*x*, *y*) represents a pixel value of the image, *w* is the mean value of the overall pixels included in the box area, *f*(*x* + *i*, *y* + *j*) represents a pixel value of the composite image inside the box area, and *f* (*i*, *j*) is the mean value of the composite image inside the box area. *K* and *L* represent the box dimensions. In the image blending stage, an advanced image blending algorithm was applied to improve the visual quality of the composite image. Figure 2b displays this process in which an overlap between a new image and the composite image is shown. For the collection of 3D quantitative surface texture parameters of all the stitched images, MATLAB^®^ 2017 software (The MathWorks, Inc., USA) was used [30,31,32,33]. The mathematical equations (Equations (4)–(19)) and description of the surface texture parameters measured in this study are presented in Table 2. The surface texture parameters obtained using the ETL system were validated with a conventional optical interferometry system (Contour GT Elite K, Bruker Nano Surfaces, Bruker UK Ltd., Coventry, U.K.) previously validated by our group with singly acquired images (*n* = 5) [3].

### 2.5. Statistical Analysis

Analysis of variance (ANOVA) (confidence limit of *p* < 0.05) was used to investigate the statistical significance between the various surface texture parameters. Moreover, linear regression and root mean square error were also calculated for the 2D line scan data points. 

## 3. Results and Discussion

**Figure 3:**
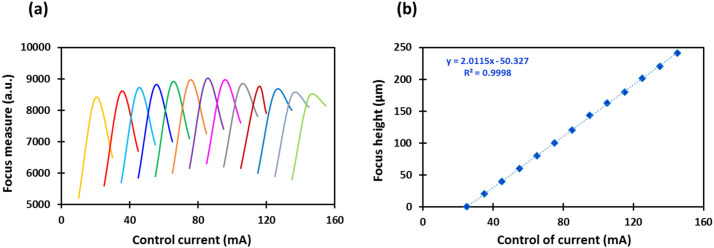
(**a**) Focus measure sweep as a function of tunable lens control current for different focus height and (**b**) focus height as a function of the tunable lens control current, calibration curve of the ETL imaging system.

To achieve high depth resolution, ensure accurate measurement of depth, and to account for and correct any influences from external factors including temperature on the ETL, calibration of the system is an essential process. The calibration process as described in Section 2.2 resulted in the Gaussian-like curves displayed in Figure 3a with each curve representing a different focus height as set on the automatic motorised vertical translation stage. The graph displays the focus measure (a.u.) achieved as calculated using the Tenengrad operator as the control current (mA) applied to the ETL which is adjusted to modify the focal length setting (Figure 3a). To ensure the focus measure accurately detects the plane of best focus, the control current (mA) required to reach the greatest focus measure (a.u.) for each focus height (µm) (peak of each curve) was plotted against its corresponding focus height (Figure 3b). This graph displayed a linear relationship between the control current applied to the ETL and the height at which the optical system focuses with a correlation coefficient of the linear regression of 0.999 indicating that the focus measure accurately detects the plane of best focus.

**Figure 4:**
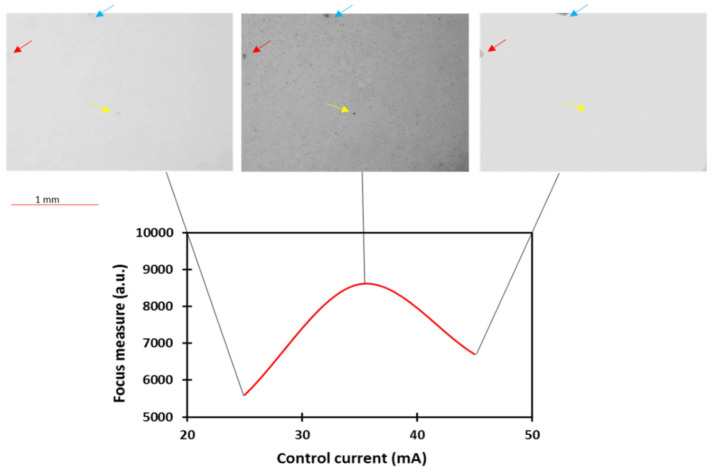
Gaussian-like curve illustrating the autofocusing ability of the system by presenting the images captured at the peak and the extreme ends of the curve.

Furthermore, the effectiveness of the Tenengrad operator as a focus measure is demonstrated in Figure 4 in which the Gaussian-like curve obtained for the 25 µm focus height is displayed along with the images captured at the peak and the extreme ends of the curve. These images reveal the most in-focus image at the peak of the curve and a decrease in focus on either side of the peak. This trend was especially evident upon visual analysis of the points of reference in each corresponding image which are highlighted with arrows in Figure 4 and display more in-focus features in the image captured at the peak of the curve compared with the images captured at either side.

**Figure 5:**
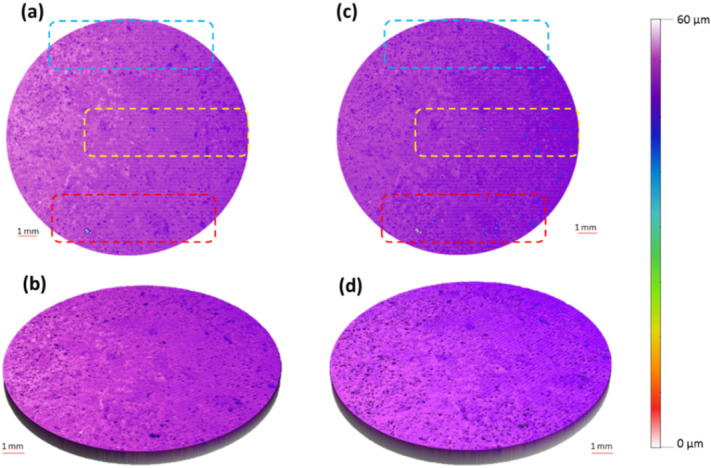
(**a**) 2D and (**b**) 3D surface texture images produced using optical profilometry. These images are adapted from [3] with permission from the publisher, Royal Society Chemistry. (**c**) 2D and (**d**) 3D surface texture images produced using the new developed ETL-based variable focus imaging system.

**Figure 6:**
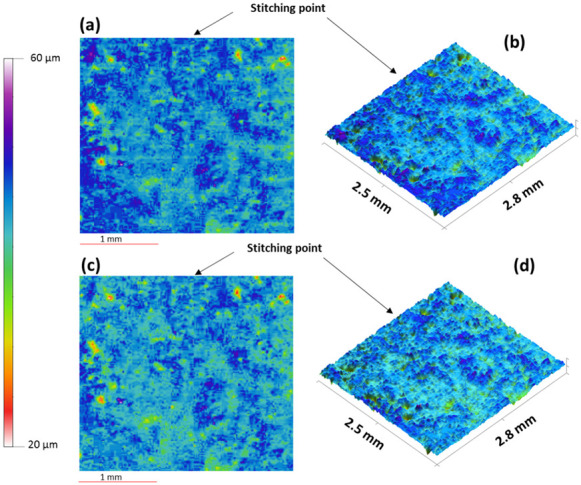
(**a**) 2D and (**b**) 3D surface texture images produced using optical profilometry, (**c**) 2D and, (**d**) 3D surface texture images produced using the newly developed ETL-based variable focus imaging system showing the zoomed area and the image-stitching points.

**Figure 7:**
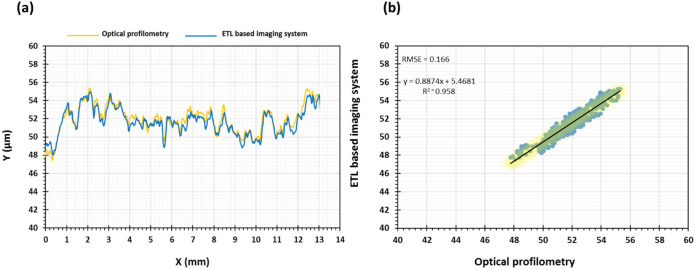
(**a**) 2D line scans of the tablets generated using a line passing through the exact centre point of the pharmaceutical tablet images generated using optical profilometry and newly developed ETL-based imaging system and (**b**) quantitative comparison by determining the root mean square error (RMSE) of the 2D line scan data points.

Qualitative surface texture analysis was conducted using the ETL system as well as optical profilometry by producing 3D images of the surface of the tablet as presented in Figure 5a–d. The images acquired from both systems were successfully stitched, as shown in Figure 6a–d, using the method reported recently by our group [3]. The overall images have not showed any discrepancy. The comparability of both techniques for the analysis of the surface texture is shown in the rectangular boxes on the tablet surface which highlight the same area of the surface in the image captured using optical profilometry (Figure 5a) and the ETL system (Figure 5c). In these images, an almost identical surface texture can be seen within the respective rectangular boxes of both techniques with a similar location, size, and intensity of valleys as demonstrated by 2D line scans (Figure 7). The 2D line scan data points were further comparatively analysed and the findings showed a good agreement in data with RMSE (root mean square error) = 0.166 and R^2^ (correlation coefficient) = 0.96, Figure 7b. Overall, these images display a smooth tablet surface with minimal peaks and an equal distribution of valleys (Figure 5a–d). However, a higher degree of valleys can be seen in the image generated by the ETL-based system as displayed by the darker spots on the tablet surface (Figure 5c,d) and 2D line scan (Figure 7). 

To further explore the comparability of both surface texture analysis techniques, quantitative analysis was also conducted by analysing surface texture parameters. The surface texture parameters explored in this study include amplitude, spacing, hybrid, and volume parameters. Amplitude parameters, which are also known as height parameters, provide analysis of vertical deviations of the roughness profile of the surface from the mean line, whereas spatial parameters focus on the direction of the plane. Hybrid parameters characterise both the height direction and the direction of the plane. Finally, volume parameters relate to the volume of material contained within specified material ratio values using the areal material ratio curve. 

**Figure 8:**
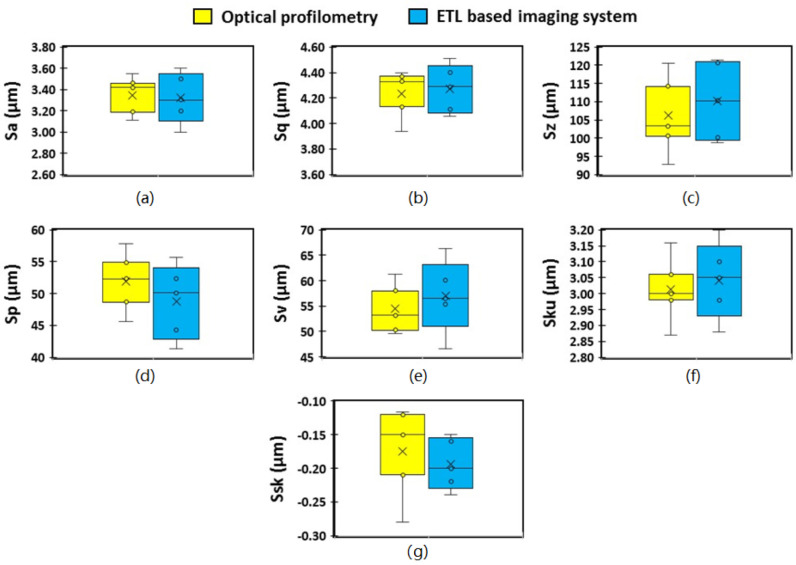
Amplitude and spacing parameters of 3D surface texture analysis. (**a**) S_a_: arithmetical average of surface; (**b**) S_q_: root mean squared height; (**c**) S_z_: deviation maximum height; (**d**) S_p_: highest peak of the surface; (**e**) S_v_: lowest valley of the surface; (**f**) S_ku_: kurtosis of height distribution; and (**g**) S_sk_: skewness of height distribution.

**Figure 9:**
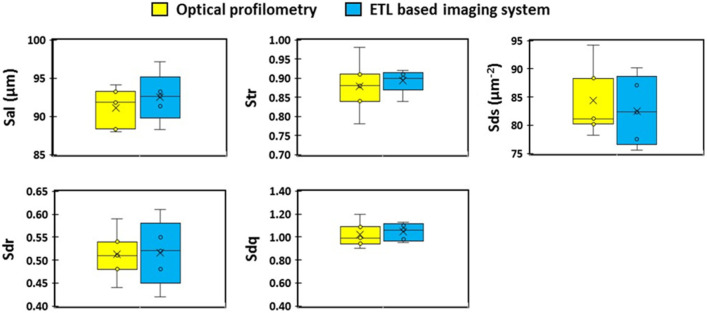
Spatial and hybrid parameters of 3D surface texture analysis. S_al_: surface autocorrelation length; S_tr_: surface aspect ratio; S_ds_: surface density summits; S_dr_: interfacial area ratio; and S_dq_: surface root mean square.

**Figure 10:**
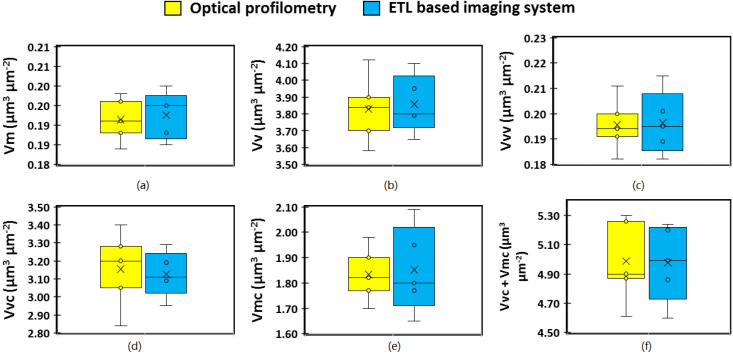
Spatial parameters of 3D surface texture analysis. (**a**) V_m_: material surface volume; (**b**) V_v_: surface void volume; (**c**) V_vv_: valley void volume; (**d**) V_vc_: core void volume; and (**e**) V_mc_: core material surface volume; (**f**) V_vc_ + V_mc_: core void plus core material surface.

Surface texture analysis was conducted using the ETL system as well as optical profilometry and results are presented in the form of box and whisker plots to allow visual comparison of the surface texture parameters obtained using both techniques (Figure 8, Figure 9 and Figure 10). The first of the amplitude parameters analysed were the arithmetical mean height (S_a_) and the root mean square height (S_q_). These measures assess the average standard deviation of the valleys and peaks in a surface profile and are typically used as a general evaluation of the surface roughness [35]. The mean S_a_ of the tablet surface obtained using the ETL system and optical profilometry was 3.35 µm and 3.32 µm, respectively, and the mean S_q_ was 4.28 µm and 4.23 µm, respectively. The maximum peak height (S_p_), which is the height of the highest peak within the defined area, and the maximum pit height (S_v_), which is the absolute value of the depth of the deepest pit within the defined area, were also analysed. The mean S_p_ and S_v_ of the surface obtained using the ETL system were 48.80 µm and 57.00 µm, respectively, and 51.86 µm and 54.48 µm, respectively, for optical profilometry. The sum of S_p_ and S_v_ were also calculated and defined as the maximum height (S_z_). The mean S_z_ of the tablet surface obtained using the ETL system and optical profilometry was 110.20 µm and 106.34 µm, respectively. Furthermore, the skewness (S_sk_), which represents the degree of symmetry of the surface heights about the mean plane, was also evaluated. The mean S_sk_ obtained using the ETL system and optical profilometry was −0.20 µm and −0.189 µm, respectively, indicating the predominance of valley structures and a height distribution skewed above the mean plane (S_sk_ < 0). The kurtosis (S_ku_), which measures the sharpness of the roughness profile, indicated either a normal distribution (S_ku_ = 3.00) or slightly skewed towards a presence of inordinately high peaks/deep valleys (S_ku_ > 3.00) as determined by the mean S_ku_ obtained using the ETL system (3.00 µm) and optical profilometry (3.044 µm). Upon visual analysis of the box and whisker plots of the amplitude parameters (Figure 8), it may be observed that the surface texture parameters obtained using the ETL system generally resulted in a slightly larger interquartile range (Figure 8a–f) indicating greater variability in the results, however, not significant.

The spatial parameters explored in this study relate to the autocorrelation function which is a measurement of how regular or periodic the surface is. This is quantified by comparing a shifted image of the surface with the original image. An autocorrelation function of 1.00 indicates that the shifted surface is identical to the original surface, whereas an autocorrelation function of −1.00 suggests that all peaks align with corresponding valleys [30]. One application of the autocorrelation function in surface texture analysis is the measurement of the autocorrelation length (S_al_) which represents the horizontal distance in the direction in which the autocorrelation function decays to a specified value (0.2 by default) the fastest. This provides a measure of the distance over the surface such that the new location will have minimal correlation with the original location. The mean S_al_ obtained used the ETL system and the optical profilometry system were 92.50 µm and 91.20 µm, respectively. Furthermore, the texture aspect ratio (S_tr_) was also measured which quantifies the spatial isotropy or directionality of the surface texture. This value is calculated by dividing the value of S_al_ by the horizontal distance in the direction of the slowest decay of autocorrelation function to the value (0.2 by default). The mean values of S_tr_ were calculated as 0.901 and 0.880 for the ETL system and optical microscopy, respectively. 

The hybrid parameters characterised in the present study include the developed interfacial area ratio (S_dr_), root mean square of the slope (S_dq_), and summit density (S_ds_). S_dr_ and S_dq_ are useful parameters for differentiating surfaces of similar amplitudes and average roughness and effectively quantify the spatial intricacy of the surface as well as the slope. The mean values of S_dr_ and S_dq_ computed using the ETL system and optical microscopy were 0.500 and 0.512, respectively, for S_dr_, and 1.00 and 1.02, respectively, for S_dq_. S_ds_, on the other hand, is a measure of the number of summits comprising the surface per unit area using a definition of a summit as 5% of S_z_. Use of the ETL system and optical profilometry produced mean S_ds_ values of 82.6 1/μm^2^ and 84.2 1/μm^2^, respectively. Both imaging techniques revealed no statistically significant difference between the methods (Figure 9).

The volume parameter defined as the material volume (V_m_) provides a measure of the volume of material of the surface from the height corresponding to a material ratio value (which may be set to any value from 0% to 100%) to the highest peak of the surface. Similarly, V_mc_ (core material volume) is the volume of material contained within the material ratio values of 10% and 80%. The mean values calculated for these two parameters using the ETL system and optical microscopy were 0.190 μm^3^/μm^2^ and 0.192 μm^3^/μm^2^, respectively, for V_m_, and 1.86 μm^3^/μm^2^ and 1.84 μm^3^/μm^2^, respectively, for V_mc_. Other volume parameters explored in the present study include the void volume (V_v_), dale void volume (V_vv_), and core void volume (V_vc_) which all provide a measure of the volume of material that would fill the surface between the chosen material ratio values [30]. The mean V_v_, V_vv_, and V_vvc_ values measured for the ETL system were 3.86 μm^3^/μm^2^, 0.198 μm^3^/μm^2^, and 3.12 μm^3^/μm^2^, respectively, and 3.82 μm^3^/μm^2^, 0.195 μm^3^/μm^2^, 3.15 μm^3^/μm^2^, respectively, for optical profilometry. Similar to the amplitude parameters, visual analysis of the box and whisker plots of the volume parameters (Figure 10) revealed slightly larger interquartile range for the parameters obtained using the ETL-based imaging system compared with optical microscopy (Figure 10a–c,e–f) indicating variability in the results, however, not statistically significant.

## 4. Conclusions

The current study successfully developed and employed an ETL variable focus imaging system for the quantitative surface texture analysis of a pharmaceutical solid dosage form (model material sample). The Tenengrad operator used to calibrate the system effectively identified the plane of best focus as demonstrated by the linear relationship between the control current applied to the ETL and the height at which the optical system focuses. The system was then successfully utilised to obtain surface texture parameters which related to the amplitude, spatial, hybrid, and volume characteristics of the surface. Moreover, validation of the ETL system against optical profilometry revealed comparable values of the parameters for both techniques with no statistically significant differences between the two. These results highlighted the potential application for ETL systems as an easily adaptable, low cost alternative non-destructive surface texture analysis method due to its enhanced DOF compared with conventional microscopy techniques including contact profilometers, AFM, coherence scanning interferometry, confocal microscopy, scanning laser microscope, and structure light scanning. It is anticipated that the benefits of this technique will have a wide-ranging impact in multiple industries as a powerful method for the surface analysis of materials including mechanical components, construction material, surgical implants, and pharmaceutical formulations.

## Figures and Tables

**Table 1:** Specifications of electrically tunable lens integrated add-on camera system.

Objective Mag	Numerical Aperture	Z-Range (µm)	Z-Resolution (µm)	Z-Axis Accuracy (%)	Z-Axis Repeatability (%)	Max. Slope
5×	0.10	640	18.5	1	0.35	90°
10×	0.25	160	3			
20×	0.45	40	1			
50×	0.80	6.4	0.25			

Data obtained from the manufacturer.

**Table 2:** Summary of 3D surface texture parameters [34].

Surface Topography Parameters		Description	Equation	
**Amplitude**	Root mean squared height (S_q_, nm)	S_q_ presents an overall measure of the texture comprising the surface.	$S_{q}=\sqrt{\frac{1}{\mathrm{MN}}\overset{N}{\underset{j=1}{\sum }}\overset{M}{\underset{i=1}{\sum }}n^{2}\left(x_{i}\;,y_{j}\right)}$	(4)
	Arithmetical average of surface (S_a_, nm)	Provides an average of the overall roughness of the surface.	$S_{a}=\frac{1}{\mathrm{MN}}\;\overset{N}{\underset{j=1}{\sum }}\overset{M}{\underset{i=1}{\sum }}|n^{2}\left(x_{i}\;,y_{j}\right)|$	(5)
	Skewness of height distribution (S_sk_)	Represents the degree of symmetry of the surface heights about the mean plane. The predominance of peaks comprising the surface is indicated by S_sk_ > 0, whereas the predominance of valley structures is indicated by S_sk_ < 0.	$S_{sk}=\frac{1}{\mathrm{MN}S_{q}^{3}}\;\overset{N}{\underset{j=1}{\sum }}\overset{M}{\underset{i=1}{\sum }}n^{3}\left(x_{i}\;,y_{j}\right)$	(6)
	Kurtosis of height distribution (S_ku_)	Reveals the presence of inordinately high peaks/deep valleys (S_ku_ > 3.00) or lack thereof (S_ku_ < 3.00) comprising the surface.	$\;\;\;\;\;\;S_{ku}=\frac{1}{\mathrm{MN}S_{q}^{4}}\;\overset{N}{\underset{j=1}{\sum }}\overset{M}{\underset{i=1}{\sum }}n^{4}\left(x_{i}\;,y_{j}\right)$	(7)
	Highest peak of the surface (S_p,_ nm)	The height of the highest point.	$\;\;\;\;\;S_{p}=\mathrm{MAX}\;\left(n_{p}\right)$	(8)
	Lowest valley of the surface (S_v,_ nm)	The depth of the lowest point expressed as a negative number.	$S_{v}=\mathrm{MIN}\;\left(n_{v}\right)$	(9)
	Deviation maximum height (S_z,_ nm)	The sum of S_p_ and S_v_.	$\;\;\;\;\;S_{z}=\left(\left|S_{p}\right|+\left|S_{v}\right|\right)$	(10)
**Spacing**	Surface density summits (S_ds_, 1/mm^2^)	Represents the number of summits per unit area comprising the surface. A summit is defined as at least 5% of S_z_.	$\;\;\;\;S_{ds}=\frac{Number\;of\;summits}{\left(M-1\right)\left(N-1\right).\;\;\Delta x\;.\;\;\Delta y}$	(11)
	Surface aspect ratio (S_tr_)	Quantifies the spatial isotropy or directionality of the surface texture. The S_tr_ parameter will be closer to 0.00, whereas a spatially isotropic texture will produce a S_tr_ of 1.00.	$\;S_{tr}=\frac{\min\;\left(\sqrt{t_{x}^{2}+t_{y}^{2}}\;\right)}{\max\;\left(\sqrt{t_{x}^{2}+t_{y}^{2}}\;\right)\;}$	(12)
	Surface autocorrelation length (S_al_,mm)	Represents the horizontal distance in the direction in which the autocorrelation function decays to a specified value (0.2 by default) the fastest. This provides a measure of the distance over the surface such that the new location will have minimal correlation with the original location.	$S_{al}=\;\min\;\left(\sqrt{t_{x}^{2}+t_{y}^{2}}\;\right)$	(13)
**Hybrid**	Surface root mean square (S_dq_)	Provides a measurement of the slopes which comprise the surface. The S_dq_ of a completely level surface is 0. When a surface has any slope, its S_dq_ value becomes larger.	$\;\;\;S_{dq}=\sqrt{\frac{1}{\left(M-1\right)\left(N-1\right)}\;\overset{N}{\underset{j=2}{\sum }}\overset{M}{\underset{i=2}{\sum }}p_{i\;,\;j}^{2}}$	(14)
	Interfacial area ratio (S_dr_, %)	Represents a percentage the additional surface area contributed by the texture as compared with the projected surface. The S_dr_ of a completely level surface is 0 and increases with the spatial intricacy of the texture.	$S_{dr}=\frac{\sum_{j=1}^{N-1}\sum_{i=1}^{M-1}A_{i,\;j}-\left(M-1\right)\left(N-1\right)\Delta x\;.\;\Delta y}{\left(M-1\right)\left(N-1\right)\Delta x\;.\;\Delta y}\;.\;100$	(15)
**Volume**	Material surface volume (V_m_, µm^3^/mm^2^)	The material volume V_m_(p) at a given material ratio p is defined as the volume of the material per unit area calculated from the (inverse) areal material ratio function.	$\;\;\;\;V_{m}=K\int_{0\%}^{p}[S_{mc}\left(q\right)-S_{mc}\;\left(q\right)]\;dq$	(16)
	Core surface volume (V_mc_, µm^3^/mm^2^)	The volume of material contained within the material ratio values of 10% and 80%.	$V_{mc}=\frac{V_{m}\left(h_{0.8\;}\right)-V_{m}\;\left(h_{0.10}\right)}{\left(M-1\right)\left(N-1\right).\Delta x.\;\Delta y}$	(17)
	Void surface volume (V_vc_, µm^3^/mm^2^)	The void volume V_v_(p) at a given material ratio p is defined as the volume of the voids per unit area calculated from the (inverse) areal material ratio function.	$V_{v}=K\overset{100\%}{\underset{p}{\int }}[S_{mc}\left(p\right)-S_{mc}\;\left(q\right)]\;dq$	(18)
	Core void surface volume (V_vc_, µm^3^/mm^2^)	The volume of space contained within the material ratio values of 10% and 80%.	$V_{vc}=\frac{V_{v}\left(h_{0.10\;}\right)-V_{m}\;\left(h_{0.8}\right)}{\left(M-1\right)\left(N-1\right).\Delta x.\;\Delta y}$	(19)
	Valley void surface volume (V_vv_, nm^3^/mm^2^)	The volume of space of the surface from the height corresponding to a material ratio value (80% by default) to the lowest valley.	$V_{vv}=\frac{V_{v}\left(h_{0.8\;}\right)}{\left(M-1\right)\left(N-1\right).\Delta x.\;\Delta y}$	(20)

## Data Availability

Data will be made available on request.
